# Influence of smoking and diet on glycated haemoglobin and 'pre-diabetes’ categorisation: a cross-sectional analysis

**DOI:** 10.1186/1471-2458-13-1013

**Published:** 2013-10-26

**Authors:** Antonis Vlassopoulos, Michael EJ Lean, Emilie Combet

**Affiliations:** 1 Human Nutrition, School of Medicine, College of Medical, Veterinary & Life Sciences, University of Glasgow, Walton Building level 4, Glasgow Royal Infirmary, G3 8SJ, Glasgow, UK, England; 2 Human Nutrition, Yorkhill Hospital, School of Medicine, College of Medical, Veterinary & Life Sciences, University of Glasgow, Glasgow, England

**Keywords:** Smoking, HbA1c, Oxidative stress, Pre-diabetes, Vegetables

## Abstract

**Background:**

The new HbA1c criteria for diagnosis of pre-diabetes have been criticised for misdiagnosis. It is possible that some elevation of HbA1c is not driven by hyperglycaemia. This study assesses associations of HbA1c, commonly assumed to relate solely to glucose concentration, with (i) smoking, a major source of reactive oxygen species (ROS) and (ii) fruit & vegetables consumption associated with improved redox status.

**Methods:**

One-way ANOVA, Chi-squared and multivariate linear regressions, adjusted for all known confounders were used to explore associations of HbA1c with self-reported smoking status and fruit & vegetables consumptions in the Scottish Health Surveys 2003–2010, among individuals without known diabetes and HbA1c < 6.5%.

**Results:**

Compared to non-smokers (n = 2831), smokers (n = 1457) were younger, consumed less fruit & vegetables, had lower physical activity levels, lower BMI, higher HbA1c and CRP (p < 0.05). HbA1c was higher in smokers by 0.25 SDs (0.08%), and 0.38 SDs higher (0.14%) in heavy smokers (>20 cigarettes/day) than non-smokers (p < 0.001 both). Smokers were twice as likely to have HbA1c in the 'pre-diabetic’ range (5.7-6.4%) (p < 0.001, adj.model). Pre-diabetes and low grade inflammation did not affect the associations. For every extra 80 g vegetable portion consumed, HbA1c was 0.03 SDs (0.01%) lower (p = 0.02), but fruit consumption did not impact on HbA1c, within the low range of consumptions in this population.

**Conclusion:**

This study adds evidence to relate smoking (an oxidative stress proxy) with protein glycation in normoglycaemic subjects, with implications for individuals exposed to ROS and for epidemiological interpretation of HbA1c.

## Background

Patients with large waists and related, potentially-reversible, metabolic features are at risk of developing type 2 diabetes and a range of chronic diseases [1]. Their specific pathologies are characterised by pro-inflammatory states and a shift in tissue redox homeostasis towards excess free-radical activity [2]. Redox status is influenced by diet and lifestyle factors, including fruit and vegetable consumption, cigarette smoking and by inflammatory disease activity [3-5]. Smoking is a well-established risk factor for diabetes, as shown by a recent meta-analysis [6], contributes to the production of reactive oxygen species (ROS) and increases production of inflammatory molecules, beta-cell dysfunction and end-organ protein damage [7-9]. On the other hand, low fruit and vegetable consumption is associated with impaired redox status in young, healthy populations [5] and diets rich in fruit and vegetable with lower concentrations of oxidative stress markers [10,11]. Individuals exposed to higher levels of oxidative stress experience greater benefits in their redox status from fruit and vegetables consumption [12].

Protein glycation is a common form of protein damage, and approximately 1%-16% of albumin is glycated in normoglycaemic blood [13,14], which has been associated with metabolic deterioration. Advanced Glycation Endproducts (AGEs) are involved in the aetiology of various chronic disease [15], especially diabetes and its vascular complications. Protein glycation levels are determined by the concentration of sugars (mainly glucose) [16,17] and dependant on the protein half life, as well as fructosamine removal through the action of fructosamine-3-phosphokinase [18]. An individual’s pro-oxidant status, however, has been speculated to be involved in later stages of the glycation reaction, leading to the formation of glycoxidation products, like pentosidine [19,20]. Moreover, glucose autoxidation, associated with increased oxidative stress, may play a role in promoting Maillard product formation, in the early glycation stages [21]. Glycated haemoglobin (HbA_1c_) is an early glycation product used diagnostically as a specific marker for glucose exposure. HbA_1c_ has become established for monitoring of glycaemic status in diabetes, as an indicator of glucose levels in the previous 90 days, and more recently its diagnosis [22]. HbA_1c_ relates strongly to tissue damage in diabetic patients and it has also been found to predict coronary heart disease (CHD) and cancer in non-diabetic individuals, even within the 'normal’ non-diabetic range (4.9 – 6.3%) [23]. The concentration of HbA_1c_ is usually assumed to relate mainly to glucose concentration in populations with similar red blood cell turn-over. Since blood glucose fluctuations are minimal within HbA_1c_ levels <5.7%, it seems possible that the relatively large variations on HbA_1c_ concentration might reflect variations in redox status, and could indicate wider protein glycation. Supporting this concept, we have shown previously, in national survey data, that HbA_1c_ in non-diabetic subjects, correlates with CHD risk factors, but that it is inversely correlated with dietary intake of fruits and vegetables, and with both dietary intake and plasma concentration of dietary antioxidants (Vitamin C, Vitamin E, Vitamin B6) [24]. We have also shown that oxidative stress is important for albumin glycation, measured as fructosamine production, at physiological glucose concentrations [25].

Since the process of glycation is non-enzymatic, it is relatively slow, so day-to-day variations are unlikely to have a major impact. This is an advantage for health surveys and screening. The Scottish Health Survey (SHS) comprises representative population-based surveys, in a population at high risk of CHD [26], conducted every 3–5 years from 1995 until 2003, and annually since 2008. We have investigated whether lifestyle drivers of redox status (cigarette smoking, fruit and vegetable consumption) are associated with protein glycation, using HbA1c as a marker of the process, in sub-samples of non-diabetic adults.

## Methods

This study is a secondary analysis of the anonymised data from the Scottish Health Survey and the Health Survey for England. Original ethical approval for the Scottish Health Surveys was granted by the Multicentre Research Ethics Committee (Scotland). As the data are in the public domain and available through the Economic and Social Data Service (ESDS), this study required no additional ethical approval ( http://www.scotland.gov.uk/Topics/Statistics/Browse/Health/scottish-health-survey/Publications).

### Subjects

Data from the 2003, 2008, 2009 and 2010 SHSs were compiled in order to create the large database used in the current analysis. The SHS is a cross-sectional nationally representative survey reporting the health and health-related behaviour of people living in private households in Scotland, using a multistage, stratified, clustered probability-sampling design. Full survey procedures are described elsewhere [27-29]; a brief summary is given here.

### General methods

During two household visits, data on demographic, economic, occupational, age, sex, general health and health related habits were collected. Weight was measured to the nearest 100 g using electronic scales. Height was measured to the nearest millimetre using a stadiometer. Body Mass Index (BMI) was calculated as weight (kg) divided by height squared (m^2^). The waist was measured at the midpoint between the lowest rib and upper margin of the iliac crest. The measurements were recorded to the nearest millimetre with at least two measurements within 5 cm combined to provide a mean [30].

### Dietary measures

Two different tools were used to assess eating habits in the Scottish Health Surveys. One, a food frequency questionnaire (FFQ) validated against weighed intakes [31], was specifically designed to assess fruit and vegetable intake. Responders were asked about the total number of portions of vegetables (fresh, frozen or canned) and vegetables in composites, salads, pulses, fruit (fresh, frozen or canned), dried fruit and fruit in composites consumed in the 24 h preceding the interview. From the available nutritional information, variables that reported total portions of vegetable, total portions of fruit and the two of them combined were selected for the current analysis.

### Smoking habits

Participants were asked to report if they were currently smoking, ex-occasional or ex-regular smokers, or never smoked. Present smoking status was classified as light (under 10 cigarettes a day), moderate (10 to under 20 cigarettes a day), heavy (20 or more cigarettes a day) and non-smoker.

### Physical activity

Frequency of participation was assessed for various domains of activity, including leisure time sports and exercise (for at least 15 minutes per occasion). The total physical activity was then categorised to 'low/medium/high’ according to the metabolic equivalents spent during each activity and the total activities reported.

### Biochemical and other measurements

Serum C-Reactive Protein (CRP) was measured by N Latex high sensitivity mono-immunoassay on the Behring Nephelometer II analyzer (coefficient of variation <6%, limit of detection 0.17 mg/l) in nmol/L (conversion factor to mg/L = 0.105) and total glycated haemoglobin (HbA_1c_) analysis was carried out in the Biochemistry Department at the RVI using the Tosoh G7 HPLC analyser, which was calibrated using Diabetes Control and Complications Trial (DCCT) standards with coefficient of variation <2.5%.

### Statistical analysis

In order to exclude undiagnosed diabetes cases, the cut-off point of HbA_1c_ ≥6.5% (48 mmol/mol) was employed [22]. Participants without diagnosed diabetes and with HbA_1c_ ≤6.5% (48 mmol/mol) were included in the analysis. Pregnant women were excluded. Individuals with HbA1c between 5.7% (39 mmol/mol) and 6.4% (46 mmol/mol) were classified as being at high risk or 'pre-diabetic’ [22]. Body mass index, mean waist circumference, age and CRP levels were used as continuous variables. CRP was also used in order to identify and exclude individuals with low grade inflammation (CRP >28.5 nmol/L or >3 mg/L) [32,33]. Data were checked for normality and homoscedasticity using the Kolmogorov-Smirnoff test, and for skewness. Parametric tests were used for data with normal and non-normal distributions since, according to the Central Limit Theorem [34], parametric tests can safely be used with skewed data when the sample size is over 500. The Student’s t-test, one way-ANOVA and χ^2^ test were used to examine the differences among groups of smoking status and fruit and vegetables intake in HbA_1c_ levels. General linear models were used to compare HbA_1c_ levels among smoking groups that were adjusted for age and sex. Multivariate linear regression was used to describe the effect of smoking and fruit and vegetables intake on HbA_1c_, after adjusting for age, sex, ethnic group, socioeconomic group, activity levels, BMI, waist circumference, CRP levels, vitamin supplementation and year of survey. Logistic regression was performed to investigate the association of smoking status and presence of increased risk for diabetes. In the case of fruit and vegetable intake, the model was adjusted to include smoking in the confounders. All analyses were performed using PASW Statistics (18.0.0) and statistical significance was taken as p < 0.05.

## Results

**Figure 1:**
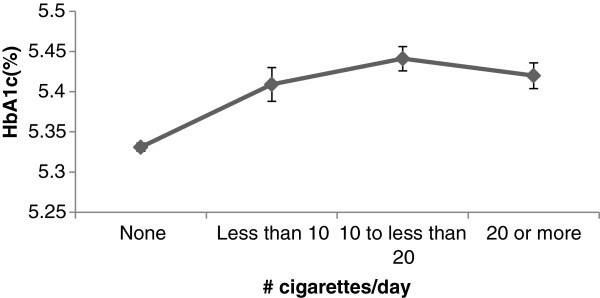
Age-sex adjusted mean (SD) of %HbA1c according to number of cigarettes/day.

**Figure 2:**
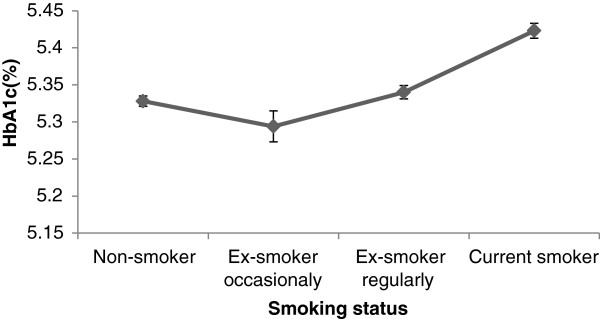
Age-sex adjusted mean (SD) of %HbA1c according to smoking status.

Age-sex adjusted %HbA1c was plotted against number of cigarette smoked per day (Figure 1) and smoking status (Figure 2). Glycated haemoglobin levels (HbA_1c_) were higher in ex-smokers and current smokers compared to non-smokers (Table 1) and this increasing trend (p < 0.001) was seen even when the age-sex adjusted means were plotted against smoking status (Figure 2). The trend remained consistent when the ex-smokers were split into those who smoked occasionally and those who smoked regularly (Table 2, Figure 2). The same analysis of age-sex adjusted HbA1c levels was conducted among groups based on number of cigarettes smoked per day, with similar results (p < 0.001) (Figure 1).

**Table 1:** Descriptive characteristics of the population in total and according to smoking status

		**Smoking status**		
	**All (n = 6120)**	**Non-smokers (n = 2831)**	**Ex-smokers (n = 1832)**	**Current smokers (n = 1457)**
Age (years)	50.5 ± 16.9 (18.0-95.0)	49.0 ± 17.2 (18–95)	56.2 ± 16.0 (19–94)*	46.1 ± 15.3 (18–91)*
Sex (% Male)	45.2	42.6	49.0*	45.4*
Social class (% High^†^)	43.2	50.6	44.2*	27.7*
Body mass index (kg/m^2^)	27.3 ± 4.8 (13.3-62.7)	27.3 ± 4.8 (15.2-53.3)	27.8 ± 4.5 (13.3-54.5)*	26.4 ± 5.1 (15.1-62.7)*
Waist circumference (mm)	91.6 ± 13.2 (54–152)	90.5 ± 13.2 (54–152)	93.8 ± 13.0 (60–152)*	89.8 ± 13.4 (58.5-147)
Physical activity (%Low)	30.7	28.1	33.7*	32.1*
HbA_1c_ (%)	5.4 ± 0.4 (2.8-6.4)	5.3 ± 0.4 (2.8-6.4)	5.4 ± 0.4 (3.8-6.4)*	5.4 ± 0.4 (2.9-6.4)*
*(mmol/mol)*	*36 ± 4.4 (30.6-46)*	*34 ± 4.4 (30.6-46)*	*36 ± 4.4 (18–46)*	*36 ± 4.4 (31.7-46)*
CRP (nmol/L)	33.3 ± 65.7 (1.9-971)	27.6 ± 55.2 (1.9-971)	36.2 ± 75.2 (1.9-95.4)*	40.9 ± 68.6 (1.9-762)*
Total fruit portions/d	2.0 ± 1.8 (0.0-20.0)	2.3 ± 1.8 (0.0-12.5)	2.2 ± 1.8 (0.0-10.5)	1.3 ± 1.7 (0.0-22.0)*
Total vegetable portions/d	1.3 ± 1.4 (0.0-22.0)	1.4 ± 1.3 (0.0-20.0)	1.4 ± 1.3 (0.0-15.3)	1.1 ± 1.3 (0.0-17.7)*
Total fruit & vegetable portions/d	3.4 ± 2.5 (0.0-31.4)	3.7 ± 2.4 (0.0-22.5)	3.6 ± 2.4 (0.0-25.3)	2.5 ± 2.4 (0.0-31.4)*

Data presented as mean ± SD (range) ^†^High social class: Professional and managerial technical; * compared to non-smokers; p < 0.05.

**Table 2:** Logistic regression for smoking status and fruit & vegetable consumption predicting high risk of diabetes

	OR*	**95% CI**	**p-value**
***Smoking status***			
*Non-smoker (contrast)*			
Ex-occasional	0.88	0.59-1.32	0.54
Ex-regular	1.11	0.92-1.34	0.26
Current smoker	***2.25***	***1.84-2.75***	***<0.001***
***# Cigarettes/day***			
*None (contrast)*			
Less than 10	***1.88***	***1.31-2.71***	***<0.001***
10 to less than 20	***2.63***	***2.04-3.39***	***<0.001***
More than 20	***2.06***	***1.57-2.71***	***<0.001***
***(Adjusted for smoking status)***			
Vegetable intake	*0.94*	*0.89-1.01*	*0.08*
Fruit intake	0.99	0.94-1.03	0.56
Fruit & vegetable intake	0.98	0.95-1.01	0.17
***(Adjusted for # cigarettes/day)***			
Vegetable intake	*0.95*	*0.89-1.01*	*0.09*
Fruit intake	0.99	0.94-1.03	0.53
Fruit & vegetable intake	0.98	0.95-1.01	0.17

*adjusted for age, sex, ethnic group, social class, physical activity level, CRP, BMI, waist circumference & year of study. # cigarettes per day: number of cigarettes per day.

Current smokers were twice as likely as non-smokers to have HbA_1c_ in the pre-diabetes range (Table 2). Lighter smokers (<10 cigarettes/day) were almost twice as likely as non-smokers to have HbA_1c_ in the pre-diabetes range (OR 1.88), while smoking 10 to 20 cigarettes per day increased the risk for HbA1c ≥5.7% (39 mmol/mol) more than 2-fold (OR 2.63) (Table 2). Being an ex-occasional or ex-regular smoker was not associated with higher chances of being classified in the high risk/pre-diabetes category of HbA_1c_.

Body mass index (BMI) differed significantly by smoking status: current smokers had a lower BMI than non-smokers when ex-smokers had a significantly higher BMI than non-smokers (Table 1). Smoking is associated with poorer social circumstances, while ex-smoking status is more frequent among those with better social circumstances (% high social class 44.2 vs. 27.7 in ex-smokers and current smokers respectively p < 0.001) (Table 1). Current smokers included younger subjects than non-smokers (46.1 ± 15.3 vs. 49.0 ± 17.2 years; p < 0.001), whereas ex-smokers included older subjects. There was no difference in waist circumference between current smokers and non-smokers, but ex-smokers had a higher mean waist circumference than non-smokers (p < 0.05). Current smokers consumed fewer portions of fruit, vegetables, and the two combined, than non-smokers and a higher proportion were classified as having low physical activity. Although ex-smokers also included more subjects classified as having low physical activity than non-smokers, they were no different for fruit and vegetable consumption. C-reactive protein concentrations were greater among smokers, both ex-smokers and current smokers having higher values than non-smokers (Table 1). Ex-smokers and current smokers had CRP levels significantly higher than the 28.5 nmol/L cut-off indicating high risk for metabolic diseases range and low-grade inflammation (p < 0.001; data not shown). In all following analysis the above mentioned variables were used as confounders.

Consumption of vegetables was associated with having marginally lower chances of having HbA_1c_ in the pre-diabetes range. This effect remained after adjustment for various confounders, using either smoking status or number of cigarettes per day (Table 2).

Multivariate linear regression showed that current smokers have higher HbA_1c_ than non-smokers, by 0.08% (0.9 mmol/mol) (equal to 0.25 times the SD of this population). Heavy smoking (20 or more cigarettes a day) is associated with a larger effect of 0.14% (1.5 mmol/mol) (equal to 0.38 times the SD of this population) greater HbA_1c_. The regression model explained ≈ 35% of the variance in HbA_1c_. From all the confounding factors employed in this study only year of survey had a significant effect on HbA_1c_ levels in the full factorial model (coefficient 0.125 ± 0.04, p < 0.001) (data not shown). HbA_1c_ levels were lower by 0.01% (0.1 mmol/mol) for each extra portion of vegetable consumed, after controlling for number of cigarettes per day. The same was not found for fruit portions per day or fruit & vegetables portion combined (Table 3).

**Table 3:** Regression analysis summary for smoking status and fruit & vegetable consumption with %HbA1c levels

***a) Non-diabetics (HbA1c < 6.5%) (n = 5425)***	Coef*	**SE**	**p-value**
Smoking status	***0.027***	***0.004***	***<0.001***
# cigarettes/day	***0.047***	***0.005***	***<0.001***
Vegetable intake^†^	***-0.009***	***0.004***	***0.008***
Fruit intake^†^	0.001	0.003	0.754
Fruit & vegetable intake^†^	-0.002	0.002	0.236
***b) High risk of diabetes (HbA1c 5.7%-6.4) (n = 1391)***			
Smoking status	0.050	0.004	0.201
# cigarettes/day	*0.010*	*0.005*	*0.060*
Vegetable intake^†^	0.001	0.004	0.947
Fruit intake^†^	0.004	0.003	0.170
Fruit & vegetable intake^†^	0.002	0.002	0.292
***c) Low risk of diabetes (HbA1c < 5.7%) (n = 4155)***			
Smoking status	***0.017***	***0.003***	***<0.001***
# cigarettes/day	***0.031***	***0.005***	***<0.001***
Vegetable intake^†^	-0.005	0.003	0.092
Fruit intake^†^	0.001	0.002	0.603
Fruit & vegetable intake^†^	-0.001	0.002	0.605
***c) Low risk of diabetes(HbA1c < 5.7%) & CRP < 28.5 nmol/L (n = 3172)***			
Smoking status	***0.014***	***0.004***	***<0.001***
# cigarettes/day	***0.029***	***0.005***	***<0.001***
Vegetable intake^†^	***-0.009***	***0.004***	***0.021***
Fruit intake^†^	0.002	0.003	0.546
Fruit & vegetable intake^†^	-0.001	0.002	0.464

*adjusted for age, sex, ethnic group, social class, physical activity level, CRP, BMI, waist circumference & year of study; ^†^adjusted for #cigarettes/day.

When individuals with HbA_1c_ in the pre-diabetes range were assessed alone, the R^2^ value of the multiple regression model for the narrow range of HbA_1c_ was only 0.06, leaving a large proportion of variance unexplained (data not shown). Thus neither vegetable nor fruit consumption had an effect on HbA_1c_ (Table 3). There was no interaction between smoking status and HbA_1c_ level (p = 0.20) but there was weak evidence for an association between number of cigarettes per day and greater HbA_1c_ levels (p = 0.06).

In order to avoid confounding effects from any mild metabolic disruptions associated with the pre-diabetic status or low-grade inflammation, the above analysis was conducted among individuals with HbA_1c_ below the 5.7% (39 mmol/mol) cut-off, and with a CRP lower than 28.5 nmol/L. Heavy smoking (10–20 cigarettes/day) was still associated with ≈ 0.1% (1.1 mmol/mol) (equal to 0.28 times the SD for this population) greater HbA1c than non-smoking (Table 3). Although vegetable consumption was significantly (inversely) associated with HbA1c among the non-diabetic individuals as a whole, the level of significance dropped for this analysis restricted to individuals with HbA1c less than 5.7% (39 mmol/mol) (p = 0.09). However, individuals with both HbA1c <5.7% (39 mmol/mol) and also CRP < 3 mmol/L had significantly lower HbA1c, by 0.01% (0.1 mmol/mol) (equal to 0.03 times the SD of this population) (p = 0.02) for every extra portion of vegetables consumed (Table 3).

## Discussion

HbA_1c_ is commonly regarded as a biomarker for blood glucose levels; however, observing the substantial variation in HbA_1c_ among non-diabetic individuals, unlikely to result from sustained differences in blood glucose, we postulated that HbA_1c_ may also reflect oxidative stress or redox status. In our analysis, we have demonstrated that smoking (a proxy for oxidative stress) is positively associated with protein glycation, as measured by HbA_1c_ levels in non-diabetic subjects. This finding adds complexity to the evidence that smoking increases the risk of developing type 2 diabetes [6,35].

Although smoking has been extensively studied as a risk factor for deteriorating diabetic status and as promoting end-organ damage in diabetic subjects, evidence on the effect of smoking on protein glycation and diabetes progression is not so clear. A large epidemiological study conducted in the USA has found similar results with smokers having a relative 7% increase in HbA_1c_ levels compared to non-smokers, in a population free of diabetes [36]. An early report of 191 hypertensive and normotensive non-diabetic subjects detected a statistically significant difference in HbA_1c_ levels between smokers and non-smokers [37]. The same was reported in a sample of 3240 healthy non-diabetic adults [38] and in 1773 middle-aged non-diabetic participants from the Potsdam cohort of EPIC [39] where smoking was associated with increased HbA_1c_ levels in both sexes. A recent Dutch study assessing the role of increased waist circumference (>88 cm for women, >102 cm for men) on protein glycation, measured by skin auto-fluorescence, in non-diabetic individuals identified current smoking as an important confounder in the association, which agrees with our data [40]. Among these diverse studies, some can be considered valid, but others have low numbers and weaknesses in design. The current study benefits from the large sample size and the numerous diabetes related variables available, in order to control for confounding factors..

In the current study we have also shown that smoking remains positively correlated with HbA_1c_ in a subsample of participants who are not affected by any metabolic derangements from pre-diabetes (with HbA_1c_ < 5.7%, 39 mmol/mol) and in a subsample with both HbA_1c_ < 5.7% (39 mmol/mol) and CRP levels < 28.5 nmol/L to exclude any influence from low-grade inflammation. This fractioning of the population was performed on the basis that individuals with HbA_1c_ levels within the prediabetic range, would already have some degree of abnormalities in glucose metabolism. These abnormalities are most likely to explain the variation in HbA1c levels in this population subgroup [41,42]. These results provide robust evidence for a true association between oxidative stress and protein glycation. In fact, we did not find evidence for the same association with smoking among the individuals with HbA_1c_ in the pre-diabetic range 5.7-6.4% (39–46 mmol/mol), probably because of smaller number of participants in this group, with a range of HbA_1c_ too narrow to detect an influence, however the main driver protein glycation in this group is likely to be their altered glycaemia and glucose level fluctuations. It seems likely that oxidative stress and redox status are more potent drivers of protein glycation on a background of normal glucose metabolism, but elevated blood glucose concentrations in the fasting and/or post-prandial states would dominate. Unfortunately lack of data on blood glucose levels did not allow for this association to be investigated in the present study. Measuring both HbA_1c_ and glucose concentrations in large epidemiological studies, together with indicators of exposure to oxidative stress and antioxidant/anti-inflammatory influences, would be of great value to discriminate these effects.

There was some evidence in the present study that vegetable consumption (a proxy for antioxidant influences on redox status) was inversely associated with HbA_1c_ levels in all non-diabetic subjects (HbA1c < 6.5%, 48 mmol/mol) and among individuals with low HbA1c (<5.7%, 39 mmol/mol) and low CRP (<28.5 nmol/L), suggesting some benefit from diets high in antioxidant rich sources. It is likely that fruit would have the same physiological effect, but the present study, in a population notorious for its low fruit consumption (and a narrow range of fruit consumptions for statistical analysis) was unable to detect an effect. Our data have shown mean intakes of vegetables to be 1.3 ± 1.4 portion per day and fruit 2.0 ± 1.8 portions per day.

In a large study of older English adults, by Bates et al. [24], both dietary antioxidant consumption and antioxidant plasma levels of non-diabetic individuals were found to have inverse associations with HbA_1c_ levels. There was also an inverse correlation between plasma vitamin C concentration and HbA_1c_. A similar inverse relationship has been found for vitamin C and vitamin E in a younger population, also suggesting that antioxidant intake and hence antioxidant capacity and redox status are playing a role in protein glycation in normoglycaemia [39]. A link between antioxidant intake and protection against protein glycation is supported by *in vitro* evidence clearly showing that antioxidants, including vitamin C, flavonoids and tocopherols reduce glycation of proteins [43,44], and pro-oxidants, such as H_2_O_2_ and lipid peroxides, drive glycation of haemoglobin [45,46]. Protein glycation and oxidation processes share common sites on the protein molecules, which supports a mechanistic relationship [47]. Oxidative stress and an associated increase in protein glycation is present not only in smokers [37] but also in chronic renal failure [48], and myocardial infarction [49]. Our study adds to an increasing body of evidence from various fields, to strengthen our hypothesis that redox status drives protein glycation in non-diabetic individuals.

This study inevitably has limitations. A cross-sectional survey design can only ever be hypothesis-generating, and does not allow for causality to be investigated. We are not claiming that oxidation is the only, or the main mechanism behind protein glycation in non-diabetic and pre-diabetic subjects, but our data do allow us to make the hypothesis that it is playing a part, and a potentially modifiable part. Using proxy measures of oxidative stress and redox status, rather than actual levels of oxidative stress markers (like isoprostanes), is an in-built limitation in this type of general-population health survey. We established the independence of pro- and anti-oxidant environmental factors by including them in the same analytical model. Specific measures could be made in future research. The lack of data on average levels of glycaemia, independent of HbA_1c_ limits the interpretation of the data. Fasting glucose or 2-hour postprandial glucose levels are seldom measured in large epidemiologic studies, and in any case one-off measurements are considered relatively unreliable as reflections of ambient blood glucose. We have explored the possible of effect of small differences in glycaemia by analyses within narrow HbA_1c_ sub-categories, and find the same associations with the indicators of redox status. It would be possible to define the independence of theses associations from those of fasting and 2-hour blood glucose in large diabetes screening datasets which also include measures to reflect redox status, but not in the present study. It is also possible that increased fruit and vegetable intake might be associated with a decreased intake of heat-processed food, a main contributor of dietary AGEs, and an increased consumption of fibre, minerals and other micronutrients. Unfortunately this association could not be investigated in this study due to the nature of the nutritional information available.

## Conclusions

This large population-based study suggests that protein glycation, indicated by HbA_1c_, is positively associated with smoking, and inversely correlated with vegetable intake. An unfavourable redox status, may thus account for some people having HbA_1c_ in the pre-diabetic range, and this mechanism may promote progression to diabetes, as well as promoting tissue damage. These results strengthen the case for the balance between antioxidant and pro-oxidant status being important in the pathogenesis of chronic diseases.

## Abbreviations

ROS: Reactive oxygen species; AGEs: Advanced glycation endproducts; HbA1c: Glycated haemoglobin; CHD: Coronary heart disease; SHS: Scottish health survey; BMI: Body mass index; FFQ: Food frequency questionnaire; CRP: C-reactive protein.

## Competing interests

The authors declare that they have no competing interests.

## Authors’ contributions

AV conducted research and analyzed data; AV, MEJL, EC wrote the paper; MEJL and EC designed research and had primary responsibility for final content. All authors read and approved the final manuscript.

## Pre-publication history

The pre-publication history for this paper can be accessed here:

http://www.biomedcentral.com/1471-2458/13/1013/prepub
